# Interplay between Natural Killer Cells and Anti-HER2 Antibodies: Perspectives for Breast Cancer Immunotherapy

**DOI:** 10.3389/fimmu.2017.01544

**Published:** 2017-11-13

**Authors:** Aura Muntasell, Mariona Cabo, Sonia Servitja, Ignasi Tusquets, María Martínez-García, Ana Rovira, Federico Rojo, Joan Albanell, Miguel López-Botet

**Affiliations:** ^1^Hospital del Mar Medical Research Institute (IMIM), Barcelona, Spain; ^2^Department of Oncology, Hospital del Mar-CIBERONC, Barcelona, Spain; ^3^Hospital Fundación Jiménez Díaz, Madrid, Spain; ^4^Univ. Pompeu Fabra, Barcelona, Spain

**Keywords:** natural killer cells, breast cancer, human epidermal growth factor receptor 2, trastuzumab, pertuzumab, antibody-dependent cell-mediated cytotoxicity, immunotherapy

## Abstract

Overexpression of the human epidermal growth factor receptor 2 (HER2) defines a subgroup of breast tumors with aggressive behavior. The addition of HER2-targeted antibodies (i.e., trastuzumab, pertuzumab) to chemotherapy significantly improves relapse-free and overall survival in patients with early-stage and advanced disease. Nonetheless, considerable proportions of patients develop resistance to treatment, highlighting the need for additional and co-adjuvant therapeutic strategies. HER2-specific antibodies can trigger natural killer (NK) cell-mediated antibody-dependent cellular cytotoxicity and indirectly enhance the development of tumor-specific T cell immunity; both mechanisms contributing to their antitumor efficacy in preclinical models. Antibody-dependent NK cell activation results in the release of cytotoxic granules as well as the secretion of pro-inflammatory cytokines (i.e., IFNγ and TNFα) and chemokines. Hence, NK cell tumor suppressive functions include direct cytolytic killing of tumor cells as well as the regulation of subsequent antitumor adaptive immunity. Albeit tumors with gene expression signatures associated to the presence of cytotoxic lymphocyte infiltrates benefit from trastuzumab-based treatment, NK cell-related biomarkers of response/resistance to HER2-specific therapeutic antibodies in breast cancer patients remain elusive. Several variables, including (i) the configuration of the patient NK cell repertoire; (ii) tumor molecular features (i.e., estrogen receptor expression); (iii) concomitant therapeutic regimens (i.e., chemotherapeutic agents, tyrosine kinase inhibitors); and (iv) evasion mechanisms developed by progressive breast tumors, have been shown to quantitatively and qualitatively influence antibody-triggered NK cell responses. In this review, we discuss possible interventions for restoring/enhancing the therapeutic activity of HER2 therapeutic antibodies by harnessing NK cell antitumor potential through combinatorial approaches, including immune checkpoint blocking/stimulatory antibodies, cytokines and toll-like receptor agonists.

## Introduction

Breast cancer is a major health-care problem worldwide, with an estimated 1.67 million women diagnosed annually.^1^ Human epidermal growth factor receptor 2 (HER2, also known as ErbB2 or HER2/neu) is a transmembrane receptor with tyrosine kinase activity, capable of activating several pro-survival intracellular signaling pathways (1). HER2 overexpression occurs in approximately 15–20% of breast tumors and is associated with aggressive disease and decreased survival (2). Addition of HER2-targeted therapeutic monoclonal antibodies (mAb) to chemotherapy improved overall survival in patients with early-stage and advanced disease (3). Currently, two complementary anti-HER2 therapeutic mAbs, trastuzumab, and pertuzumab, and the antibody-drug trastuzumab-emtansine (T-DM1) are approved for clinical use. Combination of chemotherapy with dual HER2 targeting with trastuzumab and pertuzumab are the prevailing therapeutic approaches for HER2^+^ tumors in the neoadjuvant setting and in the first-line treatment of metastatic disease; trastuzumab and lapatinib (a dual EGFR/HER2 tyrosine kinase inhibitor small molecule) can also be used in refractory patients with advanced disease (4, 5); T-DM1 has been approved for treating advanced HER2^+^ breast cancer patients with progressive disease following trastuzumab/pertuzumab and chemotherapy regimens (6). Despite significant improvement in the clinical outcome of HER2^+^ breast cancer since the introduction of these anti-HER2 drugs, there are patients with early disease that eventually relapse and disease progression inevitably occurs due to *de novo* or acquired resistance to treatment in metastatic patients (7). Potential tumor cell-intrinsic mechanisms of resistance to anti-HER2 mAb treatment have been identified, yet their clinical relevance remains uncertain (8).

All currently approved anti-HER2 mAbs are immunoglobulins (Ig) of the G1 subclass (IgG1) and, in addition to block HER2 oncogenic signaling, share the capability of triggering antitumor immune function by engaging specific receptors expressed by immune cells (FcγR family, Box 1) through their constant domain (Fc). Several publications indicate that NK and tumor-specific T lymphocytes significantly influence disease development and response to treatment with anti-HER2 mAbs (9–12). In addition to considerable data supporting the importance of T cells in immunosurveillance (9), a role for NK cell function in preventing early tumor development and metastatic spread is being increasingly appreciated (13, 14).

Box 1 **Antibody structure and FcγR family.**

Antibodies (Abs) or immunoglobulins (Ig) display two functionally different domains: a variable Fab region which determines specificity and affinity for a particular antigen and a constant region or Fc fragment which can engage a diversity of cellular receptors in immune cells. Immunoglobulins of the G subclass (IgG) can interact with distinct FcγR family members, respectively, displaying activating and inhibitory signaling capacity. Human activating FcγRs include FcγRI (CD64), FcγRIIA (CD32A), FcγRIIC (CD32C), and FcγRIIIA (CD16A), whereas FcγRIIB (CD32B) is the counterpart with inhibitory function. FcγR in mouse includes FcγRI, FcγRIII, and FcγRIV with stimulatory potential and the inhibitory FcγRIIB. Human NK cells primarily express FcγRIIIA in the absence of inhibitory FcγR; B cells exclusively express the inhibitory FcγRIIB; human dendritic cells express both the activating and the inhibitory forms of FcγRII A and B. Distinct monocyte/macrophage subpopulations have been shown to express diverse combinations of activating and inhibitory FcγR, including FcγRI, FcγRIIA, FcγRIIB, and FcγRIIIA. It is nowadays recognized that the Fc fragment of therapeutic antibodies elicits several of their effector mechanisms. Engagement of activating FcγR results in antibody-dependent cellular cytotoxicity and phagocytosis (ADCC and ADCP). With the exception of FcγRI, remaining FcγR show intermediate/low affinity for IgG and will bind to immune complexes or IgG-coated targets, resulting in receptor crosslinking and triggering of cellular responses. Human IgG2 and IgG4 isotypes display a poor interaction with FcγR whilst human IgG1 and IgG3 interact more strongly (15, 16).

In this review, current understanding of antitumor immune responses driven by anti-HER2 mAbs will be discussed from the NK cell perspective, integrating a conceptual framework for the combinatorial use of anti-HER2 antibodies and several immunotherapy approaches enhancing NK cell function/survival in breast cancer.

## Regulation of NK Cell Antitumor Function

Natural killer cells are cytotoxic members of the innate lymphocyte cell family, important in the defense against virus-infected and transformed cells. NK cell activation leads to the polarized release of cytolytic molecules, such as granzyme B and perforin stored in preformed granules, causing target cell death (14, 17, 18). NK cells can also trigger perforin-independent apoptosis by FasL- and TRAIL-mediated engagement of death-inducing receptors on target cells (19). Time-lapse imaging has revealed that a single activated NK cell can make serial contacts with multiple targets and kill an average of four tumor cells *in vitro* (20, 21). In addition, activated NK cells secrete IFNγ, TNFα, and chemokines (i.e., MIP1α, MIP1β, RANTES), boosting the recruitment of other immune effectors and the development of subsequent antitumor T cell immunity (14, 17, 18).

The importance of NK cell function for early tumor immune surveillance is supported by studies showing increased cancer risk in individuals with low NK cell activity (22), including several genetically predisposed cases (i.e., NKG2D haplotypes LNK1/LNK1) (23). On the other hand, correlation between tumor NK cell density/function and prognosis has been reported for a number of cancer types (e.g., colorectal, hepatocellular, gastric carcinomas, lung adenocarcinoma, and renal cancer), supporting their importance for metastasis control *in vivo* (13, 24, 25).

Natural killer cell activation is regulated by an array of germ-line encoded surface receptors with stimulatory or inhibitory function. NK cells use inhibitory receptors to prevent the killing of healthy cells, whereas crosslinking of activating receptors is required to initiate an immune response against transformed cells (26). NKG2D, NKp46 and NKp30, together with the co-stimulatory molecule DNAM-1, are considered the main activating receptors involved in direct tumor cell recognition (27–29). NKG2D recognizes stress-induced self-molecules, such as MICA/B and the ULBP family, upregulated in most neoplastic cell types (30); natural cytotoxicity receptors (NKp30 and NKp46) can recognize self-molecules exposed in damaged cells (i.e., BAT3, MLL5) or induced by inflammatory stimuli (i.e., B7-H6) (31, 32); and DNAM-1 specifically recognizes CD155 (PVR) and CD112 (Nectin-2), overexpressed in a variety of tumor types (33). NK cell tolerance to self depends on inhibitory receptors specific for HLA class I molecules (HLA-I), which suppress NK cell activation against healthy cells expressing normal levels of surface HLA-I. Downregulation of surface HLA-I expression, in some virus-infected and transformed cells, allows for rapid NK cell responses against these targets (34). HLA-I specific NK cell receptors comprise killer cell immunoglobulin-like receptors (KIRs; Box 2) specific for distinct sets of HLA-I molecules (HLA-A, -B, -C); the CD94/NKG2A receptor specific for the HLA-I class Ib molecule HLA-E; and the leukocyte immunoglobulin-like receptor B1 (LILRB1) interacting with a broad spectrum of HLA-I molecules, including HLA-G. KIR and NKG2 receptor families also include members with activating function which, in some cases, can interact with HLA-I molecules albeit with lower affinity than their inhibitory counterparts (i.e., KIR2DS1 and CD94/NKG2C) (18).

Box 2 **KIR receptors and their ligands.**

The KIR receptor family includes six inhibitory receptors (KIR2DL1, KIR2DL2, KIR2DL3, KIR3DL1, KIR3DL2, and KIR2DL5), six activating receptors (KIR2DS1, KIR2DS2, KIR2DS3, KIR2DS4, KIR2DS5, and KIR3DS1), and one, KIR2DL4, harbouring an ambiguous signaling motif. Inhibitory KIRs are characterized by a long cytoplasmatic tail containing ITIM motifs whereas activating KIRs have a short cytoplasmic tail and interact with DAP-12 for transducing stimulatory signals. Inhibitory KIR recognize specific epitopes on HLA-A, -B, and -C molecules, determined by polymorphisms within residues 77–83 of the α1 helix. KIR2DL2/L3 and KIR2DL1 respectively recognize the C1 and C2 epitopes, found in mutually exclusive subsets of HLA-C alleles. KIR3DL1 binds to the Bw4 epitope, carried by subsets of HLA-A and HLA-B alleles whereas KIR3DL2 interacts with the A3/11 epitope, restricted to HLA-A3 and A11 molecules. The HLA class I specificity of activating KIRs is still a matter of study. KIR2DS1 has been shown to recognize the C2-epitope, whereas KIR2DS4 can interact with groups C1 and C2 HLA-C alleles and HLA-A11. Inhibitory KIR2DL1, KIR2DL2/L3, and KIR3DL1 are highly polymorphic. Allelic variants display distinct avidity and/or specificity of the ligand-binding site, level of cell-surface expression, and signal transduction capacity. Combinations of particular KIR and HLA class I have been associated to differential susceptibility to a wide range of diseases (e.g., infectious and autoimmune syndromes) and can influence hematopoietic cell transplantation outcomes (34–36).

Besides direct recognition, FcγRIIIA (CD16A) triggers NK cell activation against antibody-opsonized cells by a mechanism known as antibody-dependent cell-mediated cytotoxicity (ADCC). NK cells and certain T lymphocyte subsets (i.e., γδ T cells) are the only immune cells expressing the activating CD16A, in the absence of other members of the FcγR family with inhibitory function (15) (Box 1). Among all activating NK cell receptors, CD16A was described as the only one capable of triggering resting NK cell activation in the absence of co-stimulation (37) and of increasing the killing frequency per NK cell (38).

Natural killer cells also express functional toll-like receptors (TLRs) (i.e., TLR2, TLR3, TLR5, TLR7/8, and TLR9), which sense the presence of microbe-associated molecular patterns (MAMPs) and damage-associated molecular patterns (DAMPs) in the microenvironment, priming NK cell effector function (39, 40).

Overall NK cell antitumor efficacy depends on the combination of activation, effector function, proliferation, and survival, all these modulated by cytokines. IL-2 and IL-15 signaling through STAT5 promotes NK cell survival as well as increased IFNγ secretion, cytotoxicity, and proliferation (41); IL-12 and IL-18 signaling through STAT4 enhances NK cell cytotoxicity and cytokine production whereas type I IFNs (IFNα/β) are strong stimuli regulating NK cell cytotoxicity through the upregulation of perforin and FasL and promoting IFNγ secretion (42, 43). Conversely, TGFβ has been shown to repress the mTOR pathway in NK cells, consequently reducing their proliferation, the abundance of various activating receptors and cytotoxic activity (44).

Similar to T lymphocytes, NK cells can express several activation-induced co-receptors with stimulatory (e.g., CD137, OX40, NKp44) or inhibitory (e.g., PD1, TIGIT) function which constitute yet another layer of regulatory elements for NK cell activation (45).

## NK Cell-Mediated ADCC as Mechanism of Action of Anti-HER2 Antibodies

**Figure 1:**
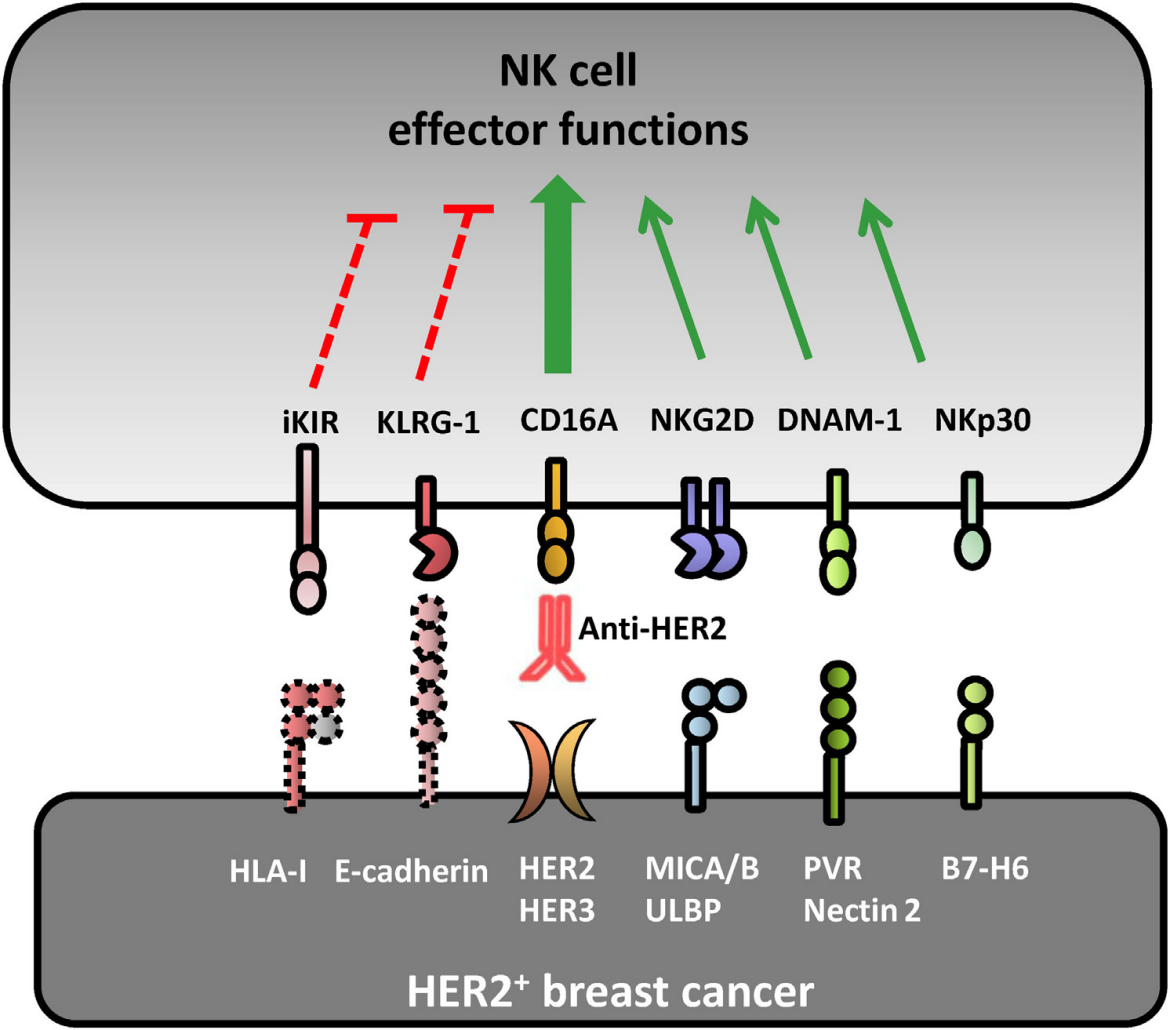
Receptor–ligand pairs involved in natural killer (NK) cell recognition of HER2^+^ breast cancer cell lines. Several receptor–ligand pairs are involved in the crosstalk between breast cancer (BC) cells and NK lymphocytes. Natural cytotoxicity against HER2^+^ BC is mainly driven by NKG2D, DNAM-1, and NKp30 activating receptors upon interacting with their cognate ligands MICA/B, PVR/Nectin-2, and B7-H6, respectively. Human epidermal growth factor receptor 2 (HER2)-dependent downregulation of surface HLA-I expression impairs KIR-mediated inhibition facilitating NK cell recognition of BC cell lines. Anti-HER2 therapeutic monoclonal antibodies elicit a strong NK cell-mediated antibody-dependent cell-mediated cytotoxicity response against HER2^+^ BC cells upon interaction with the activating CD16A receptor. E-cadherin expression can be recognized by KLRG1 inhibitory receptor expressed by some NK cell subsets, modulating their direct and antibody-dependent cytotoxicity.

Natural killer cell recognition of HER2-overexpressing target cells involves a number of receptors that can determine natural cytotoxicity upon direct recognition or influence the magnitude of ADCC in the presence of HER2-specific mAbs (Figure 1).

HER2 signaling was shown to downregulate HLA-I and promote MICA and MICB protein expression in breast cancer cell lines *in vitro*, enhancing their susceptibility to NKG2D-mediated NK cell recognition and elimination (46–49). Indeed, an inverse relationship between HER2 and HLA-I expression was corroborated by immunohistochemistry (50) and concordant mRNA signatures in HER2^+^ tumors (51). As a matter of fact, gene expression signatures associated to cytotoxic lymphocytes are enriched in the stroma of good prognosis HER2^+^ tumors (52), suggesting that HER2^+^ breast carcinomas might be permissive to NK cell infiltration, at least at early stages of tumor development.

Anti-HER2 therapeutic mAbs introduced a novel ground by which NK cells could contribute to breast tumor control. Preclinical and clinical observations indicate that triggering of NK cell-mediated ADCC is one of the mechanisms accounting for anti-HER2 mAb therapeutic activity (53). Trastuzumab activity against xenografted tumors was severely attenuated in mice deficient in activating FcγR receptors (54) and trastuzumab F(ab′)_2_ fragments (lacking Fc domain) showed marginal antitumor activity *in vivo* despite retaining their anti-proliferative and pro-apoptotic effects *in vitro* (55). More precisely, NK cell depletion abolished anti-HER2 mAb therapeutic activity in preclinical mouse models of HER2^+^ breast cancer (56–59).

Indirect evidence also points to a significant contribution of NK cells to the clinical success of anti-HER2 mAb in breast cancer patients. Numbers of tumor-infiltrating leukocytes, particularly NK cells, were reported to increase after trastuzumab-docetaxel (60, 61) and T-DM1 treatment (62), suggesting that anti-HER2 mAb promoted NK cell tumor homing or *in situ* expansion. Remarkably, immune–gene expression signatures reflecting an increased recruitment of activated NK and T cells in breast tumors (i.e., CD8A, CD247, CD3D, GZMA) have been shown to be predictive of clinical benefit from preoperative and adjuvant trastuzumab-based treatment (52, 63, 64). On the other hand, peripheral blood NK cells from patients undergoing complete or partial remission upon trastuzumab plus chemotherapy displayed high ADCC activity in *in vitro* lysis assays, whereas impaired NK cell-mediated ADCC responses correlated with therapy failure (65, 66). Of note, a number of factors, including the disparity in markers used for precise NK cell enumeration in tumor sections (e.g., CD57, CD56, GzmB) and the absence of standardized functional read-outs, have hindered the development of NK cell-related biomarkers of response to anti-HER2 therapeutic mAbs.

## Variables Potentially Modulating NK Cell-Mediated ADCC in HER2^+^ Breast Cancer

**Figure 2:**
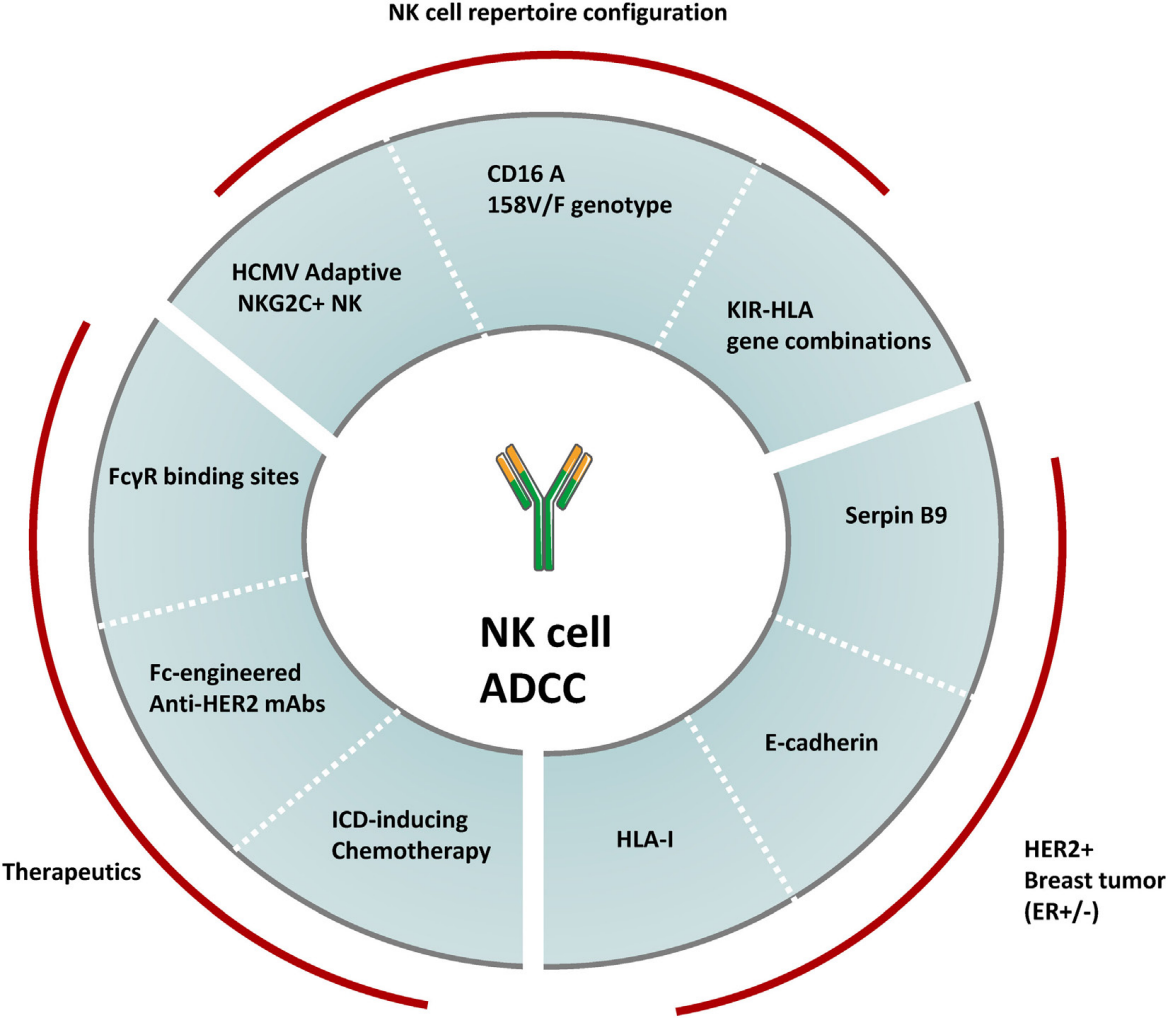
Variables modulating NK cell-mediated ADCC against HER2^+^ breast cancer. The overall magnitude of NK cell-mediated ADCC induced by anti-HER2 therapeutic monoclonal antibodies can be modulated by several factors including the configuration of the human NK cell repertoire, the heterogeneity in HER2^+^ breast tumor molecular subtypes and differences in treatment regimens. Factors such as specific KIR-HLA combinations, the CD16A 158V/F genotype and the prevalence of human cytomegalovirus (HCMV) adaptive NKG2C^+^ NK cells have been shown to modulate the overall NK cell-mediated ADCC potential. A number of tumor molecular features associated to estrogen receptor (ER) co-expression (e.g., expression of Serpin B9, E-cadherin, and HLA-I) can also modulate NK cell-mediated ADCC responses. Finally, the NK cell effector potential against HER2^+^ breast cancer is also modulated by therapeutic regimens, including the type of HER2-targeting drugs and the combined chemotherapy agents.

The specific contribution of NK cell-mediated ADCC on the clinical benefit of anti-HER2 mAb in breast cancer patients could be modulated by several NK cell-, tumor cell- and therapy-related variables (Figure 2).

### Influence of the NK Cell Repertoire Configuration on the Magnitude of ADCC

In healthy adults, approximately 90% of NK cells in peripheral blood belong to the cytotoxic CD56^dim^CD16^+^ subpopulation capable of developing ADCC responses. A second major NK cell subpopulation, defined by a CD56^bright^ phenotype and the absence of the CD16A receptor, accounts for 10% of circulating NK cells, prevails in secondary lymphoid organs and lacks ADCC potential. Among CD56^dim^CD16^+^ NK cells, several subsets displaying different NK cell receptor combinations are found at variable frequencies. Interindividual variability on the NK cell receptor repertoire is dictated by genetic and environmental factors. Major genetic factors include KIR and HLA-I genotypes. The KIR locus contains a variable number of genes, which together with their allelic diversity, determine the existence of a substantial number of distinct KIR haplotypes distributed in the world population (34, 35). KIR genes are stochastically expressed along NK cell differentiation, generating NK cell clones with discrete KIR combinations (34, 67). Only NK cell clones expressing at least one inhibitory receptor specific for self-HLA-I achieve functional maturity. Thus, KIR–HLA-I interactions contribute to set the overall functional potential in the patient NK cell repertoire (68–70). Whether certain KIR–HLA-I gene combinations can modulate the efficacy of anti-HER2 mAbs in breast cancer patients remains unaddressed, yet associations between distinct paired KIR/KIR-ligands and clinical responses to other tumor antigen-specific mAbs, such as anti-GD2 dinutuximab, anti-CD20 rituximab, or anti-EGFR cetuximab have been reported (71, 72).

Another genetic factor known to modulate antibody-dependent NK cell activation is the CD16A (FcγRIIIA) 158V/F allelic dimorphism encoding for two receptor variants harboring either a phenylalanine (F) or valine (V) at amino acid position 158 in the receptor IgG-binding domain (73). Presence of a V residue defines receptors with high affinity for IgG1 (73). An initial association between the high affinity CD16A 158V/V genotype and complete clinical responses to trastuzumab-based treatment was described in a retrospective analysis of a small cohort of metastatic breast cancer patients (74). Nonetheless, the association of CD16A 158V/F dimorphism with time to relapse and overall survival in larger patient cohorts receiving trastuzumab in adjuvancy remains controversial (75–77). Possible caveats accounting for the different results in these studies have been discussed in critical reviews (9, 78).

Environmental factors challenging the immune system, such as autoimmune or chronic inflammatory diseases and infections, can also shape the configuration of the NK cell compartment. In this regard, infection by human cytomegalovirus (HCMV) promotes, in some individuals, a persistent adaptive expansion of long-lived NK cells hallmarked by the elevated expression of the CD94/NKG2C activating receptor (79–81). Adaptive NKG2C^+^ NK cells are functionally mature and have been associated with the control of HCMV infection in kidney transplant recipients (82, 83) as well as with protection from leukemia relapse upon hematopoietic stem cell transplantation (84). Remarkably, NKG2C^+^ NK cells display enhanced effector function upon antibody-driven recognition of virus-infected targets and rituximab-coated B lymphoblastoid cell lines *in vitro* (85–87).

### Influence of HER2 Breast Cancer Molecular Subtypes on NK Cell-Mediated ADCC

Hormone receptor status differentiates two HER2^+^ breast tumor subgroups with distinct pathological response rate and overall survival upon anti-HER2 mAb treatment (88). The benefit of anti-HER2 therapy is highest in estrogen receptor (ER)-negative tumors and progressively decreases in tumors with increased ER expression (89). Globally, many immune parameters in HER2^+^ breast tumors (i.e., TILs, CD8^+^ infiltrate) are inversely correlated with ER or progesterone receptor expression (90), and it is tempting to propose a possible relationship between decreased clinical benefit of ER^+^ tumors to anti-HER2 mAbs and their increased resistance to NK cell-mediated ADCC. E-cadherin expression associated to ER^+^ breast carcinomas (91, 92) dampens trastuzumab-dependent ADCC through its specific interaction with the inhibitory killer cell lectin-like receptor G1 (KLRG1) on NK cells in preclinical *in vitro* and *in vivo* models (93, 94). Remarkably, resistance to trastuzumab-based treatment has been associated to E-cadherin expression in tumors from patients with HER2^+^ metastatic breast cancer (94). In addition, estrogens regulate the transcription of SerpinB9/proteinase inhibitor 9, a granzyme B inhibitor shown to decrease the susceptibility of ER^+^ breast cancer cells to NK and CD8^+^ T cell cytotoxicity *in vitro* (95, 96). Estrogens also upregulate HLA-I transcription through a cis-regulatory element in breast cancer cell lines (97–99), potentially modulating their susceptibility to NK cell-mediated ADCC. The relationship between ER and HLA-I expression has been confirmed by HLA-I immunohistochemical score in ER^+^/HER2^+^ as compared to ER^−^/HER2^+^ tumors (90). Whether other molecular features underlying breast carcinoma heterogeneity (e.g., mutations in PI3K, PTEN, p53, or p95HER2) (8) may modulate the susceptibility to NK cell-mediated ADCC remains uncertain.

### Therapeutic Strategies Modulating NK Cell-Mediated ADCC

HER2 dual targeting with trastuzumab in combination with pertuzumab is nowadays the gold standard therapeutic approach for HER2^+^ breast cancer in the neoadjuvant setting and in the first-line treatment of metastatic disease. Patients that have progressed to prior trastuzumab, pertuzumab, and T-DM1 are treated with lapatinib. Both therapeutic strategies augment the coating of HER2^+^ tumors with IgG1 increasing the possibilities for NK cell-mediated ADCC antitumor responses. Simultaneous binding of pertuzumab and trastuzumab to HER2 increases the density of FcγR binding sites on HER2^+^ tumors; lapatinib does so, by preventing HER2 phosphorylation and internalization, hence increasing HER2 availability for trastuzumab (100–103).

Genetic engineering of the antibody Fc domain for optimizing FcγR engagement is one of the current strategies explored for enhancing the clinical success of several tumor antigen-specific mAbs (104). Margetuximab, an Fc-optimized HER2-specific mAb in clinical development, displayed increased binding to CD16A and elicited enhanced ADCC in breast cancer preclinical models (105). Promising single-agent activity of margetuximab has been recently reported for HER2^+^ breast and gastric cancer patients with advanced disease (106). Results of an ongoing two-arm open-label Phase 3 clinical trial in front of trastuzumab (NCT02492711) will reveal whether margetuximab displays superior efficacy, particularly for patients homozygous for the CD16A 158F/F low affinity genotype, in whom margetuximab showed the highest enhancement of NK cell-mediated ADCC in preclinical studies (105).

In addition to anti-HER2 mAbs, concomitant chemotherapy regimens may significantly impact on NK cell ADCC responses. Several chemotherapeutic agents currently combined or sequentially administered with anti-HER2 mAbs (i.e., anthracyclines, cyclophosphamide, taxanes) elicit a particular type of apoptosis, known as immunogenic cell death (ICD), that is accompanied by the coordinated release of DAMPs (e.g., ATP, and HMGB1) (107). DAMPs released along ICD activate a panel of pattern-recognition receptors (e.g., TLRs, P2RX7) and promote type I IFN release from cancer cells and the secretion of pro-inflammatory cytokines by immune cells (107, 108). Among DAMPs released along ICD, HMGB1 has been shown to enhance NK cell activation and recruitment to the tumor in a TLR2/4-dependent manner in preclinical models (109, 110) whereas type I IFNs have been shown to be necessary for the therapeutic efficacy of anti-HER2 mAb in MMTV-ErbB-2 transgenic mouse model (58). Indeed, a type I IFN signature predicted clinical responses to anthracycline-based chemotherapy in several independent cohorts of patients with breast cancer (108). In addition, *in vitro* treatment with anthracyclines and taxanes enhanced anti-HER2 mAb-induced ADCC by promoting endoplasmic reticulum-stress and the upregulation of NKG2D-ligands in breast carcinoma cells (111–113). Contravening the traditional view that chemotherapeutic drugs suppress patient immunity, anthracyclines- and taxanes-based treatments associated to enhanced NK cell function in breast cancer patients (60, 113–116).

On the whole, studies integrating information on the patient NK cell repertoire, NK cell receptor ligands on tumor cells and concomitant treatments might shed light on putative resistance mechanisms to anti-HER2 mAbs in HER2^+^ breast cancer patients.

## NK Cell-Mediated ADCC and the Vaccine-Like Effect Induced by Anti-HER2 mAbs

Recent data highlight the importance of a vaccine-like effect by which antitumor mAb treatment facilitates the subsequent development of tumor-specific T cell responses, contributing to tumor elimination (117, 118). Antigen-presenting cells [i.e., macrophages and dendritic cells (DC)] use FcγR-mediated phagocytosis of immune complexes for enhancing tumor antigen processing and presentation, which can result in tumor-specific T-cell immunity (16, 117–119). Certainly, several evidences support the importance of antitumor T cell immunity for the clinical benefit of anti-HER2 mAb in breast cancer patients (115, 120–124).

Tumor cell cytotoxicity and cytokine/chemokine secretion upon antibody-dependent NK cell activation might directly and indirectly contribute to the vaccine-like effect induced by HER2-specific mAbs. On one hand, NK cell tumor cytolytic activity increases the availability of tumor antigen-containing immune complexes for antigen processing and presentation by DC and macrophages present in the tumor microenvironment. Independently of anti-HER2 mAbs, NK cell-DC crosstalk, involving cell–cell contacts and IFNγ, has been shown to prime DC polarization for IL-12 secretion, enhancing cross-presentation of tumor antigens to cytotoxic CD8^+^ T cells and the polarization of tumor-specific Th1 CD4^+^ T cells in preclinical models (59, 125–129). Moreover, activated NK cells are presumably capable of selectively killing immature DC while sparing activated DC, owing to their differential levels of surface HLA-I expression (130), thus selecting for immunogenic DC, effective inducers of antitumor T cells (127, 131). In patients, evidence of the participation of NK cell-mediated DC “editing” to the development of tumor-specific T cell immunity remains elusive. On the other hand, anti-HER2 mAb-dependent NK cell activation results in the production of IFNγ and chemokines (MIP1α, MCP-1, RANTES, IL-8) (132), which might contribute to the recruitment and functional polarization of myeloid and T cells with antitumor potential. Noteworthy, coordinated NK and tumor-specific T cell responses have been detected in HER2^+^ breast cancer patients achieving pathological complete response to trastuzumab (133).

## NK Cell Evasion in Breast Cancer

Neoplastic cells can develop a wide array of strategies to subvert NK cell recognition and cytotoxic function along tumor evolution (134, 135). Indeed, NK cell selective pressure contributes to tumor immunoediting leading to the emergence of evasive tumor cell clones (136–139). Generally, strategies hijacking NK cell function can be grouped into four categories: (i) shedding of ligands for NK cell activating receptors from tumor cells which act as decoy molecules leading to NK cell functional impairment (e.g., MICA/B, B7-H6) (140, 141); (ii) upregulation of ligands for inhibitory NK cell receptors (e.g., HLA-I molecules; PD-L1) (142, 143); (iii) dysregulated expression of molecules conferring resistance to NK cell-mediated cytotoxicity (e.g., Bcl-2; Bcl-xL, cFLIP, caspase 8, Fas) (144); and (iv) immune suppressive cytokines (e.g., IL-10, TGFβ) and metabolites (e.g., PGE2, adenosine) leading to NK cell dysfunction (135, 145).

Among all these strategies, increased levels of soluble MICA/B have been described in breast cancer patients (146) as well as overexpression of HLA-E, HLA-G in HER2^+^ tumors as determined by immunohistochemistry (147, 148). In addition, Fas downregulation in breast tumors has been correlated with shorter patient survival (149). Hence, several NK cell-evading strategies operating along breast tumor progression may hamper the antitumor efficacy of anti-HER2 mAbs.

In concert with the development of an immune suppressive microenvironment in the progressing tumor, NK lymphocytes infiltrating advanced and metastatic breast carcinomas displayed an altered phenotype and reduced cytotoxic potential (150). According to data from distinct tumor types, NK cell infiltrates included high proportions of CD56^bright^ NK cells with increased expression of inhibitory CD94/NKG2A and decreased expression of activating NKp30, NKG2D, and DNAM-1 receptors (150). NK cells isolated from breast tumors also displayed reduced degranulation and IFNγ and TNFα production upon direct or antibody-dependent activation (150). Likewise, stratification of breast cancer patients by local and invasive disease, evidenced a progressive functional impairment of circulating NK cells associated to phenotypic alterations (150). Remarkably, CD16 expression on circulating NK cells was rather preserved, and cytotoxic responses induced by trastuzumab against the HER2^+^ breast cancer cell line SKBR3 were only affected at low trastuzumab doses in NK cells from patients with locally advanced or metastatic tumors (51, 151).

## Enhancing NK Cell-Mediated ADCC through Immunotherapy in HER2 Breast Cancer

**Figure 3:**
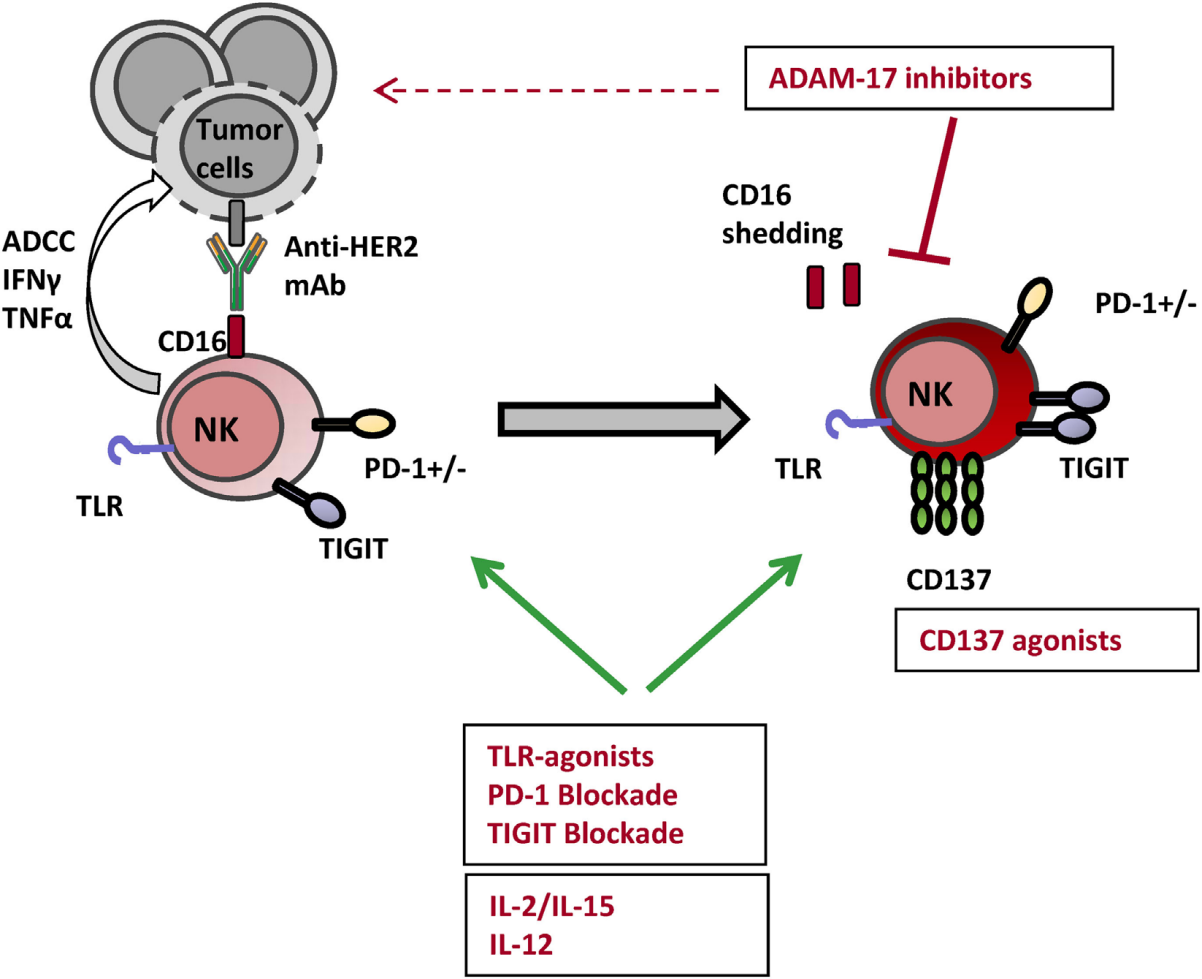
Actionable NK cell checkpoints for enhancing anti-HER2 mAb-induced ADCC responses. Several strategies can be tackled for harnessing NK cell ADCC responses with the objective of enhancing the clinical efficacy of anti-HER2 mAbs. Toll-like receptor (TLR) agonists and cytokines such as IL-2, IL-15, and IL-12 have been shown to lower NK cell activation threshold and enhance their effector potential. Among immune checkpoint modulators targeting surface receptors, anti-TIGIT and anti-PD-1 blocking mAbs as well as anti-CD137 agonist mAbs enhance NK cell-mediated ADCC and survival. Impeding CD16 shedding with A disintegrin and metalloproteinase 17 (ADAM17) inhibitors can be yet another strategy amplifying NK cell-mediated ADCC triggered by HER2-specific therapeutic mAbs.

Only two mAbs, trastuzumab and pertuzumab and the antibody-drug conjugate T-DM1, are currently approved for breast cancer treatment. Strengthening NK cell-mediated ADCC responses through immunotherapy appears a suitable option for enhancing their clinical efficacy (45, 152, 153). In the following paragraphs, several approaches will be discussed based on data referring to HER2^+^ breast cancer (Figure 3).

### Immunomodulatory mAbs Targeting Constitutive and Inducible Receptors in NK Cells

Several observations provide the rationale for combinatorial approaches including anti-HER2 mAbs and antibodies targeting surface NK cell receptors or co-receptors with activating and inhibitory function, termed immune checkpoints modulators. Nonetheless and despite promising results in preclinical models, clinical trials combining anti-HER2 mAbs and immune checkpoint-targeting antibodies are currently lacking.

IFNγ secretion by NK cells has been shown to contribute to the tumor adaptive immune resistance response (154) by upregulating the expression of HLA-I and PD-L1 in HER2^+^ breast cancer cells *in vitro* and *in vivo* (58, 155, 156). HLA-I and PD-L1 can be, respectively, recognized by KIR, CD94/NKG2A, LILRB1, and PD-1 inhibitory receptors, modulating the subsequent recognition of transformed cells by NK and T lymphocytes.

Blocking mAbs targeting HLA-I-specific inhibitory receptors with constitutive expression in NK cells include an anti-NKG2A (monalizumab, IPH2201) and an anti-KIR (lirilumab, IPH2101, BMS-986015) (45). Both antibodies are currently in early clinical development being tested for safety and efficacy mostly for the treatment of hematological malignancies.^2^ No clinical trials combining anti-HER2 mAbs and blocking agents targeting KIR or CD94/NKG2A are being developed, yet the safety and early efficacy of monalizumab and cetuximab (anti-EGFR) combination is being tested for the treatment of head and neck cancer (NCT02643550). Of note, an unexpected NK cell unresponsiveness consequent to treatment with lirilumab associated with treatment limited clinical efficacy in multiple myeloma patients (157, 158) warned about the undesired consequences of chronic targeting of HLA-I-specific NK cell receptors.

An alternative strategy, with unprecedented success as standalone treatment for several cancer types, is the blockade of the immune cell inhibitory PD-1/PD-L1 axis. Though generally considered a T cell co-receptor, PD-1 is also expressed by human exhausted NK cells (159) and circulating PD-1^+^ NK cell subpopulations were reported to be enriched in individuals with chronic viral infections as well as in cancer patients (159–161). PD-1^+^ expression is restricted to mature CD56^dim^CD16^+^ NK cells and interferes with activation *via* NKp30, NKp46, or CD16 receptors (159). PD-L1 expression was preferentially detected in HER2^+^ breast tumors showing a strong cytotoxic local immune response (162) and the numbers of PD-1^+^ tumor-infiltrating lymphocytes were associated with poor prognosis in HER2^+^ breast cancer (163, 164). Remarkably, combination of HER2-specific mAbs with blocking antibodies targeting the PD-1/PD-L1 showed greater efficacy in preclinical models (58, 62). These observations support the suitability of combining anti-HER2 mAbs with immunotherapy targeting the PD1/PD-L1 axis. Several clinical trials assessing the benefit of mAbs targeting the PD1–PD1-L axis as monotherapy or in combination with chemotherapy, radiotherapy or hormone therapy are currently being developed for ER^+^ or triple-negative breast tumors (see text footnote 2); likewise, combinatorial approaches with anti-HER2 mAbs are warranted.

TIGIT, a nectin-binding inhibitory co-receptor showing overlapping ligand specificity with the activating DNAM-1, is another inducible receptor with the capacity to modulate NK cell ADCC responses (165, 166). Both receptors recognize CD155 (also known as PVR) and CD112 (also known as Nectin-2), ubiquitous cell-adhesion molecules (167) overexpressed in HER2^+^ breast cancer cell lines (51). Besides CD8^+^ T cells, TIGIT is preferentially expressed on CD16^+^ NK cells and upregulated upon activation *via* ADCC (168, 169). TIGIT blockade has been shown to enhance trastuzumab-triggered antitumor response by human NK cells *in vitro* (169). Currently, an anti-TIGIT blocking mAb (OMP-313M32) is in early clinical development being tested for safety as standalone treatment in patients with locally advanced or metastatic solid tumors (NCT03119428).

Another immune checkpoint shown to synergize with anti-HER2 mAb in xenotransplant models of breast cancer is CD137 (58, 170). CD137 (4-1BB; TNFRSF9) is a co-stimulatory receptor induced in activated leukocytes, originally described for its capacity to enhance antitumor T cell responses (171, 172). CD137 expression following CD16 ligation has been shown in murine and human NK cells (173) and CD137 upregulation has been well documented on *ex vivo* circulating NK cells from breast and head and neck cancer patients upon tumor antigen-specific mAb infusion (170, 174). Two agonistic anti-CD137 mAb are currently in clinical development (urelumab and utomilumab), being tested alone or in combination with anti-PD-1 mAbs in advanced solid and hematologic tumors (45).

Of note, since NK and some T lymphocyte subsets share many receptor/ligand pairs involved in their functional regulation (e.g., PD-1, TIGIT, 4-1BB/CD137, and CD94/NKG2A), combinations between anti-HER2 therapeutic mAbs and distinct immune checkpoint modulators would promote antitumor immunity by dual targeting T and NK cell functional exhaustion.

### Anti-HER2 mAb Combination with Cytokines

Several attempts to potentiate NK cell antitumor function by systemic treatment with recombinant cytokines have also been carried out. Besides their effects on T cells, IL-2, and IL-15 signaling through STAT 5 enhance NK cell antitumor function (41, 175, 176).

IL-2 enhanced NK cell-mediated ADCC triggered by anti-HER2 mAb against breast cancer cell lines *in vitro* and *in vivo* (177, 178). However, clinical trials including combined administration of IL-2 with trastuzumab did not show improved disease outcome in metastatic HER2^+^ breast cancer patients (179, 180). Caveats of systemic IL-2 administration include treatment-associated toxicity, its rapid clearance *in vivo* and IL-2 pro-tumor effects through the concurrent activation of CD4^+^ regulatory T cells. Nonetheless, low-dose IL-2 is currently included in a number of clinical trials to support cellular adoptive approaches with combined infusions of NK cells and trastuzumab in HER2^+^ breast cancer patients (NCT02030561, NCT02843126).

IL-15 is an essential cytokine for human NK cell homeostasis; nonetheless, early clinical assays including systemic IL-15 were withdrawn due to concurrent adverse events and dose-limiting toxicities (181). Similarly, IL-15 enhanced the antitumor activity of trastuzumab, yet causing fatal side effects in a humanized tumor mice model (182). Current research efforts include the development of cytokine variants with extended *in vivo* half-life and targeted action on precise lymphocyte subsets and tumor sites (i.e., engineered IL-2 “superkine,” IL-15Rα Sushi-Fc fusion protein; IL-15 tri and tetraspecific killer engagers) (183–186).

IL-12 has been shown to enhance the antitumor actions of trastuzumab *via* the enhancement of NK cell IFN-γ production in mouse models (56, 57). In a clinical trial in which IL-12 was combined with trastuzumab and paclitaxel, increased levels of IFN-γ and several chemokines were detected in sera from patients with clinical benefit, but not in patients with progressive disease (187). Currently, two clinical trials are ongoing including IL-12 and trastuzumab combined treatment (NCT00004074, NCT00028535). Preclinical studies are focused on the development of approaches for targeting cytokine expression in the tumor site to avoid toxicities associated to systemic treatment (i.e., tumor-targeting immunocytokines, gene therapy with loco-regional injections of cytokine-encoding plasmid) (188).

### Immunotherapy with TLR Ligands

Toll-like receptor TLR ligands have been shown to improve both the quality and the magnitude of host antitumor innate and adaptive immune responses (189). TLR2, TLR3, TLR8, and TLR9 agonists have been shown to prime NK cell effector function (39, 40) and to synergize with anti-HER2 mAb therapy in a type I and II IFNs-, NK-, and CD8^+^ T cell-dependent manner in preclinical models (190–192). In the context of breast cancer, TLR ligands are being tested as adjuvants in diverse HER2-peptide vaccination strategies (i.e., TLR9-ligand CpG ODN in NCT00640861; TLR7 agonist imiquimod in NCT02276300; AS15 mixture in NCT02364492, NCT00058526, NCT00140738; TLR3 agonist Hiltonol in NCT01532960), including trastuzumab in some instances (i.e., the TLR9-ligand PF03512676-CpG 7909 or agatolimod-: NCT03512676, NCT00043394, NCT00031278). Strategies for delivering TLR agonists into the tumor site would likely potentiate NK cell-mediated ADCC synergizing with anti-HER2 mAbs antitumor function.

### ADAM Inhibitors

One of the consequences of CD16-mediated NK cell activation is the shedding of CD16 extracellular domain by the induced action of the A disintegrin and metalloproteinase 17 (ADAM17), thus limiting subsequent CD16A receptor engagement and NK cell activation (193). Intriguingly, ADAM10 (with constitutive activity) and ADAM17 (inducible) also control the release of ligands for EGFR/HER receptors (194) and promote the shedding of B7-H6 and MICA/B ectodomains, amplified and overexpressed in breast tumors (195, 196) limiting NKp30- and NKG2D-mediated NK cell activation (140). In fact, ADAM10 and ADAM17 levels have been associated with poor responses and shorter relapse-free survival after trastuzumab treatment (197, 198). In this scenario, inhibition of ADAM17/10 could improve NK cell-mediated ADCC triggered by anti-HER2 mAb, preventing CD16 and B7-H6 shedding as well as enhancing HER2 surface availability. ADAM17 specific inhibitor prevented CD16 shedding and improved NK cell-mediated ADCC responses *in vitro* (199). Two clinical trials tested the combination of an ADAM17 inhibitor (INCB7839) with trastuzumab (NCT01254136, NCT00864175) yet the development of the compound was suspended by the sponsor corporation and no results were published. Currently, the possibility of enhancing NK cell-mediated ADCC by combining ADAM17 inhibitor (INCB7839) and tumor antigen-specific antibodies is being tested in combination with rituximab (NCT02141451).

## Concluding Remarks

Activation of NK cell effector functions by anti-HER2 therapeutic antibodies can directly contribute to tumor control by their direct cytolytic activity against transformed cells, but also indirectly by their effects on the tumor microenvironment, eventually favoring the development of antitumor adaptive immunity. Multiple strategies are being developed for enhancing NK cell-mediated antibody-dependent antitumor activity, while simultaneously targeting other immune cells which contribute to the control of tumor growth and spreading. Understanding which variables underlie breast cancer heterogeneity in terms of lymphocyte infiltration and susceptibility to immune surveillance, as well as how the heterogeneity in the NK cell repertoire can influence on the clinical benefit of HER2-targeting mAbs, will aid in the design of tailored strategies to broaden their therapeutic window.

## Author Contributions

All authors have actively contributed to build up the conceptual framework developed in this review and revised the draft written by AM.

## Conflict of Interest Statement

Authors individually declare that the research was conducted in the absence of any commercial or financial relationship that could be construed as a potential conflict of interest.

## Footnotes

^1^http://globocan.iarc.fr/old/FactSheets/cancers/breast-new.asp.

^2^http://clinicaltrials.gov
